# Simultaneous Classification of Both Mental Workload and Stress Level Suitable for an Online Passive Brain–Computer Interface

**DOI:** 10.3390/s22020535

**Published:** 2022-01-11

**Authors:** Mahsa Bagheri, Sarah D. Power

**Affiliations:** 1 Faculty of Engineering and Applied Science, Memorial University of Newfoundland, St. John’s, NL A1C 5S7, Canada; mbagheri@mun.ca; 2 Faculty of Medicine, Memorial University of Newfoundland, St. John’s, NL A1C 5S7, Canada

**Keywords:** passive brain–computer interface, online BCI, electroencephalography (EEG), mental workload, stress, transfer learning, classification

## Abstract

Research studies on EEG-based mental workload detection for a passive BCI generally focus on classifying cognitive states associated with the performance of tasks at different levels of difficulty, with no other aspects of the user’s mental state considered. However, in real-life situations, different aspects of the user’s state such as their cognitive (e.g., level of mental workload) and affective (e.g., level of stress/anxiety) states will often change simultaneously, and performance of a BCI system designed considering just one state may be unreliable. Moreover, multiple mental states may be relevant to the purposes of the BCI—for example both mental workload and stress level might be related to an aircraft pilot’s risk of error—and the simultaneous prediction of states may be critical in maximizing the practical effectiveness of real-life online BCI systems. In this study we investigated the feasibility of performing simultaneous classification of mental workload and stress level in an online passive BCI. We investigated both subject-specific and cross-subject classification approaches, the latter with and without the application of a transfer learning technique to align the distributions of data from the training and test subjects. Using cross-subject classification with transfer learning in a simulated online analysis, we obtained accuracies of 77.5 ± 6.9% and 84.1 ± 5.9%, across 18 participants for mental workload and stress level detection, respectively.

## 1. Introduction

Passive brain–computer interfaces (BCI) are systems that aim to monitor the mental state (either cognitive or affective) of a user and exploit this information to adapt an ongoing human–machine interaction in some useful way [1,2]. For example, a passive BCI could improve road safety by first detecting states of extreme fatigue or drowsiness in the driver of a car or transport truck and then using this information to initiate alarms or other safety measures to help avoid an accident. In passive BCI systems, information on the user’s state is extracted from neural signals collected using an appropriate functional imaging modality. Electroencephalography (EEG), which measures the electrical activity of the brain, is considered as perhaps the most promising modality given its portability, high temporal resolution, non-invasiveness, and relatively low cost [3].

One highly impactful potential application of passive BCI is the monitoring of mental workload in high-risk and safety-critical occupations such as air traffic controllers, pilots, and other industrial operators, where incidents of human error can have severe consequences. Such a system would detect potentially dangerous states of high cognitive demand or overload and initiate measures to help mitigate the risk of error (e.g., have the system temporarily automate some tasks). Mental workload detection also has potential value in other domains, including gaming [4], adaptive training/learning [5,6,7], and user interface design applications [8], to enhance and personalize user experience. Because of its potential usefulness in a range of applications, mental workload detection via EEG is a very active and expanding field, with a large number of published studies (e.g., [9,10,11,12,13,14,15,16,17,18,19,20,21,22,23,24,25,26,27]).

Mental workload is defined as the perceived relationship between an individual’s total mental processing capability and the amount required by the task at hand; perceived workload is higher when the task requirements are closer to the individual’s capability [28]. Mental workload is influenced by a number of factors including the properties of the task being performed (e.g., difficulty), the task environment (e.g., noisy, distracting), and the characteristics of the individual performing the task (e.g., cognitive capacity, level of training in the task, mood) [29]. Despite this, studies of EEG-based mental workload detection generally consider workload exclusively in terms of the task demands as manipulated through the variation in task difficulty and do not consider any other factors. We argue that consideration of the user’s affective state, and specifically their stress/anxiety level, is of particular importance. Stress is known to negatively impact cognitive efficiency and performance [30] as well as decision making, especially when performing unfamiliar tasks [31]. Thus, a more complete picture of the user’s cognitive state as it relates to their potential task performance and risk for error would include a combination of both the difficulty of the task they are performing and their level of anxiety/stress. An individual performing a difficult task very calmly as opposed to performing the task while experiencing a significant amount of anxiety are two very different scenarios with very different associated risks, and the environmental adaptation strategies implemented by the passive BCI should be very different in these cases. Thus, we argue that it is very important to develop a passive BCI that is able to detect both the difficulty of the task the individual is performing and their stress/anxiety level simultaneously, in a sort of “two-dimensional” measure of cognitive load. Detecting two mental states simultaneously via EEG, with each state confounding the other, is a challenge that had not previously been addressed in the literature.

In our previous work, we found that while both states (i.e., stress, and mental workload due to task difficulty) could indeed be simultaneously classified at levels significantly exceeding chance, variation in each state negatively affected the classification of the other by EEG [25]. Expanding on this work, we aimed to improve the classification of each of the two states in the presence of variation in the other state; we proposed a majority vote-based approach that modestly but significantly increased the classification accuracy of each state [26]. While the results of these studies were promising, the preliminary analyses were conducted offline and not in a manner suitable for real-time classification. It is therefore not clear how the results will translate to a practical, online passive BCI system for the simultaneous monitoring of mental workload level and stress.

In this paper, we advance our previous work by implementing simultaneous classification of mental workload level and stress using analysis techniques entirely compatible with online classification. We investigate both entirely subject-specific and cross-subject (i.e., using training data from subjects other than the test subject) classification approaches. In the latter case, a transfer learning technique recently proposed by [32] is employed to improve classification accuracy. Because of restrictions on research involving human participants arising due to the COVID-19 global pandemic, we were unable to conduct a separate experiment for this work, and instead used the data previously collected and reported in [25,26]. While the analysis for the current paper was by necessity performed offline, the methods used simulate exactly an online classification scenario and are completely suitable for direct online implementation.

In the following sections we describe our experimental methods, provide more details about the classification approaches investigated, and report and discuss our findings.

## 2. Materials and Methods

### 2.1. Participants

Eighteen right-handed subjects (mean age: 26 ± 8 years: 11 men) were recruited for the experiment. Participants had to be between the ages of 18 and 65 years, have normal or corrected-to-normal vision and hearing, and have no history of neurological disease, disorder or injury, or cognitive impairment. Subjects were asked to refrain from smoking, ingesting alcohol or caffeine, or exercising within 4 h of the start of the experimental session.

At the start of the session, participants were first fully informed about the details of the experiment, and then signed a consent form freely indicating their willingness to participate. The study was conducted in accordance with the Declaration of Helsinki, and the protocol was approved by the Interdisciplinary Committee on Ethics in Human Research at Memorial University of Newfoundland (approval #20190461-EN).

### 2.2. Physiological Signal Acquisition

A 64-channel electrode system (ActiCHamp, Brain Products, GmBH, Gilching, Germany) was used to collect the EEG data. The position of the electrodes on the scalp was based on the standardized International 10-10 System for EEG electrode placement. FCz served as the reference electrode. The impedance of recording electrodes was kept below 10 kΩ to ensure signal quality. Three-lead electrocardiogram (ECG) was used to capture cardiac activity. The sampling frequency for both the EEG and ECG data was 500 Hz.

### 2.3. Experimental Procedure

The experimental protocol described in this section has been previously reported in [25,26] and is described again here for the convenience of the reader. In the experiment, different levels of mental workload (Easy and Difficult) were induced simultaneously with different affective states (Relaxed and Stressed) to produce four different mental state conditions: low workload/relaxed, low workload/stressed, high workload/relaxed, and high workload/stressed.

#### 2.3.1. Structure of Experimental Session

**Figure 1:**
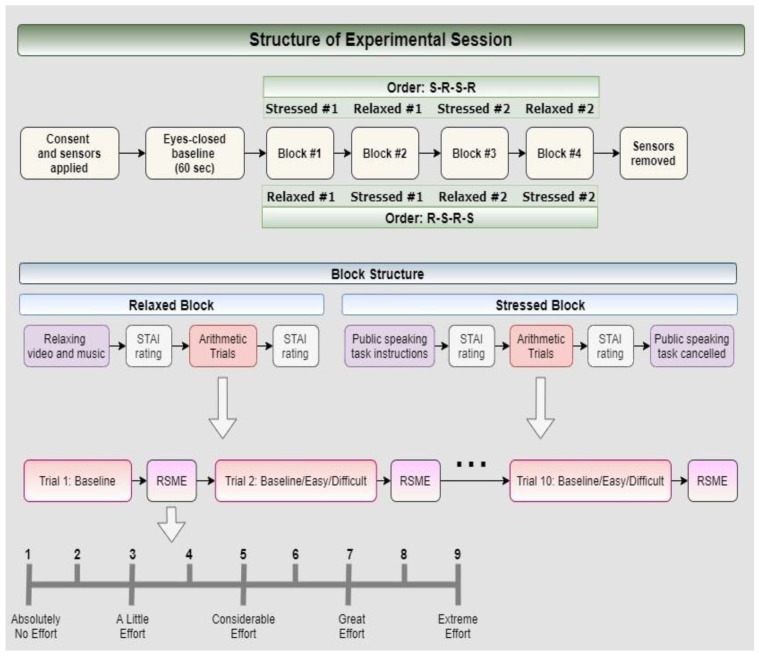
Structure of the experimental session.

Participants first completed a one-minute eyes-closed baseline trial at the beginning of the experiment. Then, they were asked to complete four blocks of tasks alternating between a Relaxed (R) and a Stressed (S) state. Half of the participants started the experiment with a Relaxed block (i.e., block order was R1-S1-R2-S2), while the other half began the experiment with a Stressed block (i.e., block order was S1-R1-S2-R2). The Relaxed and Stressed states were induced at the beginning of each block (see Section 2.3.2 and Section 2.3.3 for state induction protocols). Each block then comprised ten trials: four eyes-open baseline trials, three “Easy” arithmetic trials (low workload), and three “Difficult” arithmetic trials (high workload). Trial order was different for each of the four blocks. In all cases, no two consecutive trials were of the same difficulty level. The structure of the experimental session is depicted in Figure 1.

#### 2.3.2. Mental Workload Induction

The different levels of mental workload were induced using mental arithmetic trials of varying difficulty. The arithmetic trials involved solving arithmetic problems in the form

(1) A modulus B=C

where *A*, *B*, and *C* were positive integers. A single equation in this form appeared on the screen at the beginning of each trial, and participants had to indicate if the equation was correct or incorrect by pressing either the right arrow button or the left arrow button, respectively, on a standard keyboard. The equation remained on the screen until the participant responded with a key press, then the next equation would appear. The subject’s performance in the task (i.e., % correct = # correct/# completed) appeared in the right corner of the screen and was updated after each response. The number of equations completed varied from trial to trial depending on the pace of the participant’s responses.

Participants completed arithmetic trials at two levels of difficulty, “Easy” (i.e., low workload) and “Difficult” (i.e., high workload). Since divisibility rules for 2, 5, and 10 are relatively simple, *B* in Equation (1) was restricted to either of these numbers for the “Easy” trials (e.g., “35 mod 2 = 1” which would be correct, “46 mod 5 = 3” which would be incorrect). On the other hand, there are not simple divisibility rules for 3, 4, 6 and 9; therefore, *B* in Equation (1) was restricted to either 3, 4, 6 or 9 for the “Difficult” trials (e.g., “77 mod 9 = 5” which would be correct, “32 mod 6 = 4” which would be incorrect). For all trials, *A* in Equation (1) was a two digit positive integer.

Note that it was a priority in this work that the induction of the two states—mental workload (as related to task difficulty) and stress/anxiety—was conducted as independently as possible. Therefore, the arithmetic task was carefully designed with the objective that distinct levels of mental workload would be produced in the low and high conditions, but that the difficulty of the high condition would not be so extreme that it would itself induce stress/anxiety in the participants. Following each arithmetic trial, the participant was asked to rate their perceived level of mental effort during the trial using a modified version of the Rating Scale of Mental Effort (RSME) [33] (see Figure 1).

Along with the arithmetic trials, baseline trials were also conducted in which participants were asked to sit quietly for the duration of the trial with their eyes focused on a cross that displayed in the center of the screen.

#### 2.3.3. Affective State Induction

In order to induce a relaxed state for the Relaxed blocks, a 2-min video with relaxing imagery and music was played at the beginning of the block. Participants then completed a block of arithmetic trials. They were instructed to try their best in completing the arithmetic trials while knowing their performance would not be saved or compared to others.

In order to induce a stressed state for the Stressed blocks, the participants were told that they would first have to complete a set of arithmetic trials of varying difficulty, followed by a 10-min public speaking presentation that would be judged by a panel consisting of colleagues of the principal investigator (SP). They were told that they would have 10 min to prepare the presentation, on a topic chosen by the experimenter, and that the presentation would be recorded for future analysis. The stress induction protocol was based on the Trier Social Stress Task (TSST) [34], but in this case it is the anticipation of the public speaking task that is used to induce stress during the arithmetic trials. To further induce stress during the trials, the participants were also told that their performance was very important and would be recorded and compared to the other participants. In the first stressed block (S1), after completing the arithmetic trials the participants were told that they were not required to do the public speaking task after all “due to their performance and the physiological data obtained during the arithmetic trials”. This caused them to return to a relaxed state for the next Relaxed block. For the second Stressed block (S2), participants were again told that they would have to perform arithmetic trials followed by a public speaking task, but that this time they would be required to do the public speaking task regardless of their performance in the arithmetic task. To remove any doubt the participant may have had about this, they were allowed to overhear a phone call made prior to the arithmetic trials by the experimenter to the principal investigator asking the judging panel to arrive in 15 min, in time for the public speaking task. After the completion of the arithmetic trials of the second Stressed block, the participants were again told that the public speaking would not be necessary. At this point, the participants were told why the deception regarding the public speaking task was used, as a tool to induce stress during the arithmetic trials.

Immediately before and after the arithmetic trials in each block, form Y-1 of the State-Trait Anxiety Inventory (STAI) questionnaire [35] was completed by participants to capture self-reported stress levels. Note that for the Stressed blocks, the form was completed immediately after participants were told about the public speaking task, and then again just before they were told they did not actually have to do it.

When the participant had completed all four blocks, the EEG and ECG electrodes were removed, and the session ended.

### 2.4. Validation of Stress and Workload Induction

The detailed results of the analysis performed to validate the stress and workload induction protocols were first reported in [25]; a summary of the most relevant results is given here.

To validate the affective state induction protocol, we analyzed both objective (heart rate) and subjective (STAI questionnaire ratings) measures of stress. Two-way repeated measures ANOVAs with two within-subject factors (workload level and stress condition) revealed that both heart rate ($F_{\left(1,17\right)}$ = 4.43; *p* = 0.05) and STAI rating ($F_{\left(1,17\right)}$ = 14.66; *p* = 0.001) were significantly lower in the “Relaxed” state as compared to the “Stressed” state [25]. Furthermore, a separate two-way repeated measure ANOVA with two within-subject factors (stress condition and block number) showed no significant effect of “Block” on STAI scores ($F_{\left(1,17\right)}$ = 3.63; *p* = 0.07). Together, these results suggest that our affective state induction protocol was effective in inducing stressed and relaxed states in both the first and second blocks of each condition [25]. Moreover, it is noteworthy that there was no significant effect of workload level ($F_{\left(1,17\right)}$ = 0.3; *p* = 0.58) and no interaction effect of workload level and affective state ($F_{\left(1,17\right)}$ = 0.25; *p* = 0.62) on heart rate, indicating that, as desired, stress/anxiety was not induced due to the Difficult task condition.

To validate the mental workload induction protocol, we analyzed both objective (task response accuracy and response time) and subjective (RSME ratings) measures of workload level. Two-way repeated measures ANOVAs with two within-subject factors (workload level and stress condition) indicated that both the RSME score ($F_{\left(1,17\right)}$ = 82.14; *p* < 0.001) and the average response time ($F_{\left(1,17\right)}$ = 21.33; *p* < 0.001) were significantly lower, and the response accuracy was significantly higher ($F_{\left(1,17\right)}$ = 16.07; *p* ≤ 0.001), in the Easy condition than the Difficult condition, indicating that the protocol was effective in inducing distinct levels of perceived mental workload/effort in the “Easy” versus the “Difficult” task conditions [25]. As desired, there was no significant main effect of affective state on any of the three workload measures ($F_{\left(1,17\right)}$ < 2.54; *p* ≥ 0.13) [25].

It is worth noting that even though care was taken to induce mental workload and stress as independently of one another as possible, and the data suggests that in general we were successful in this goal, we cannot be sure that the two states are not mixed up to a certain point in some subjects due to the individual differences between people.

## 3. Data Analysis

### 3.1. EEG-Based Classification of Mental Workload Level and Affective State

In this paper, we performed simultaneous EEG-based classification of mental workload level (i.e., “Easy” vs. “Difficult”) and affective state (i.e., “Relaxed” vs. “Stressed”) using methods directly transferable to an online BCI. The classification was performed separately on each participant’s data.

An entirely subject-specific as well as two cross-subject methods were investigated. In the entirely subject-specific case, the training data consisted of the first two blocks of data (one Relaxed and one Stressed) from the test participant, and the test data consisted of their final two blocks of data (one Relaxed and one Stressed). In the cross-subject cases, the training data consisted of the first two blocks of data from the test participant as well as all data from the 17 other subjects, while the test data again consisted of the final two blocks of data from the test participant. In the second cross-subject case, a transfer learning (TL) method recently proposed by [32] was used to match the distributions of the training and test data. In all cases, the “baseline” trials were omitted from analysis.

Prior to classification, the following preprocessing and feature extraction steps were applied to the training and test data.

### 3.2. EEG Preprocessing

First, a band-pass filter was employed with low and high cut-off frequencies of 1 Hz and 50 Hz. The data were then downsampled from 500 Hz to 256 Hz, and Artifact Subspace Reconstruction (ASR) was used to reject signal segments containing EMG and motion artifacts.

To simulate an online application of the system, these preprocessing steps were applied to individual windowed segments (4 s length, 50% overlap) of the test data, in the order in which they were collected (i.e., chronological order). Specifically, each raw segment passed through all steps of the preprocessing, feature calculation, and classification, and predictions for both mental workload and stress level were made for that segment, before the next segment was processed. This is different from offline processing, where, typically, each step of the analysis (preprocessing, feature calculation, and classification) are performed on all test samples before proceeding to the next step. The analysis was performed on the same PC on which the pre-recorded EEG data were stored.

### 3.3. EEG Signal Feature Calculation

Frequency domain features were computed to represent the characteristics of the EEG signals. Power signals in seven common frequency bands were extracted via the filter-Hilbert method, and average power was calculated over 4-s epochs with a sliding window with 50% overlap. The individual alpha frequency (IAF) [36] was calculated using the one-minute eyes-closed baseline trial collected at the beginning of the experimental session and used to define the seven EEG frequency bands for each participant as follows: Delta (IAF−8 to IAF−6), Theta (IAF−6 to IAF−4), Alpha1 (IAF−4 to IAF−2), Alpha2 (IAF−2 to IAF), Alpha3 (IAF to IAF+2), Beta (IAF+2 to IAF+20), and Gamma (IAF+20 to IAF+30).

For the affective state classification problem (Stressed vs. Relaxed) all EEG channels were included, which resulted in a total of 63 electrodes × 7 frequency bands = 441 features. For the workload level classification problem (Easy vs. Difficult), since response frequency was greater for the Easy condition, we excluded the electrodes over the motor and sensorimotor brain regions (Cz, C1–C6, CPz, CP1–CP6) to ensure that incidental differences in the motor requirements of the Easy and Difficult conditions did not contribute to the classification. This resulted in a total of 49 electrodes × 7 frequency bands = 343 features for the workload level classification.

### 3.4. Classification

For both the mental workload level classification and the affective state classification, three classification paradigms were investigated and compared, as described below.

1. Subject-specific paradigm: For each target subject, the classifier was trained on the subject’s first two blocks of data and tested on the subject’s final two blocks. A regularized Linear Discriminant Analysis (LDA) algorithm was used for classification [37].
2. Cross-subject without TL paradigm: For each target subject, the classifier was trained on the subject’s first two blocks of data combined with all data from the other 17 subjects and tested on the subject’s final two blocks of data. No transfer learning algorithm was applied. A regularized LDA algorithm was used for classification [37].
3. Cross-subject with TL paradigm: For each target subject, the classifier was trained on the subject’s first two blocks of data combined with all data from the other 17 subjects and tested on the subject’s final two blocks of data. The InstanceEasyTL transfer learning method was applied to reject the differences between data coming from different subjects; this method was originally proposed in [32] and is described in detail in the section below.

For each of the above classification problems, a majority vote-based approach proposed in our previous work to improve the classification of each state in the presence of the other was employed [26]. Specifically, in each case three classifiers were trained and the final predicted class was the result of a majority vote among the three. For the case of mental workload level classification, the first classifier was trained on data from the Relaxed state only, the second classifier was trained on data from the Stressed state only, and the third classifier was trained on data from both states. Similarly, for the affective state classification, the first classifier was trained on data from the Easy condition only, the second classifier was trained on data from the Difficult condition only, and the third classifier was trained on data from both conditions.

Note that the final two blocks of data consisted of 12 trials, each of duration 67 s, for a total of 804 s of data. With 4 s epochs with 50% overlap, this yielded a classification every 2 s, for a total of 402 test samples per subject.

#### InstanceEasyTL Algorithm

EEG-based mental state classification typically requires a large amount of labeled data for training. Given the non-stationarity of EEG data within a subject, as well as significant intersubject variation, using data previously collected from other subjects, or even from the same subject on a previous day, often leads to poor classification accuracy [38,39]. This is a challenge for developing real-world online BCI systems since impractically long calibration sessions are needed in order to gather sufficient data from the user to train the classifiers immediately before each use. Recently, transfer learning (TL) methods have been applied to mitigate this issue [40]. TL models are efficient methodologies that aim at transferring the previously extracted features from a labeled domain to a similar but different domain with limited or no labels, to perform some specific decision tasks on the different domain [41,42,43,44]. With such methods, for a given mental state classification task, EEG signals previously recorded from a set of subjects can be used to train a BCI system that will be suitable for any user, whether their data is in the training set or not. This is called cross-subject classification.

**Figure 2:**
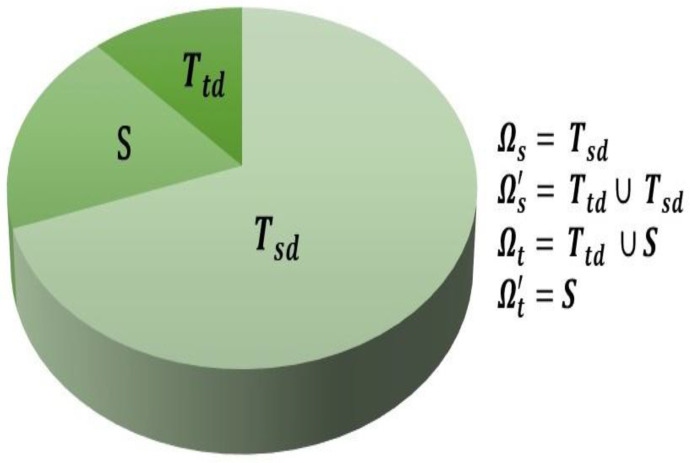
The original and new source and target domains. ${Ω}_{s}$ and ${Ω}_{t}$ are the original source and target domains, respectively, while ${Ω}_{s}^{'}$ and ${Ω}_{t}^{'}$ represent the new source and target domains used in the InstanceEasyTL algorithm, respectively. Figure adapted from [32].

In this paper, we apply the InstanceEasyTL method, which was recently proposed in [32] to improve cross-subject EEG-based fatigue detection, to our objective of simultaneous classification of both mental workload level and stress. InstanceEasyTL is based on the EasyTL method [43], which was developed for image classification applications but is adapted to work on EEG data where there are often much larger differences in the target (test subject) and source (training subjects) domain distributions. Indeed, in [32], this method led to an increase of more than 15% compared to other existing transfer learning methods such as transfer component analysis (TCA) [45], geodesic flow kernel (GFK) [46], and domain-adversarial neural networks (DANN) [47]. InstanceEasyTL is based on a “strategy of alignment with certain weights to align EEG samples collected from both source and target domains” [32]. To do this, InstanceEasyTL takes some EEG samples from the target domain ${Ω}_{t}$ (i.e., the test subject’s data) and combines it with all data from the original source domain ${Ω}_{s}$ (i.e., the training subjects’ data) to form a new source domain for training. This increases the amount of data available for training without increasing the amount of time needed for calibration prior to BCI use. As shown in Figure 2, the test subject’s data (the initial target domain, ${Ω}_{t}$) is divided into two parts: S and $T_{td}$; $T_{td}$ is added to the training subjects’ data (the initial source domain, ${Ω}_{s}$, also called $T_{sd}$ here) to form the new source domain ${Ω}_{s}^{'}$, and S is the data from the test participant that ultimately undergoes classification (i.e., the new target domain, ${Ω}_{t}^{'}$).

For our data set, we take the first 50% of the test subject’s data (coming from the first two blocks) as $T_{td}$ and combine it with the other 17 subjects’ data (${Ω}_{s}$) to create the new source domain ${Ω}_{s}^{'}$, which is used for training the classifier. We then take the final 50% of the test subject’s data (coming from the final two blocks) as S, the test data.

A complete mathematical description of the InstanceEasyTL algorithm was first proposed in [32]; for the convenience of the reader the main steps are summarized below.

For the first iteration, t, of the algorithm, initial weights are assigned to the data from both the training source domain, $T_{sd}$, and training target domain, $T_{td}$, (both from ${Ω}_{s}^{'}$) via Equation (2) [32]

(2) $$W_{sd}^{1}={⋃}_{i=1}^{n_{s}}w_{sd}^{i}\;W_{td}^{1}={⋃}_{i=n_{s}+1}^{n_{s}+m}w_{td}^{i}\;W^{1}=W_{sd}^{1}\cup \;W_{td}^{1}$$

here, $n_{s}$ and m are the number of samples in $T_{sd}$ and $T_{td}$, respectively, and $w_{sd}^{i}$ and $w_{td}^{i}$ are the weights for the i-th sample from $T_{sd}$ and $T_{td}$, respectively. Initial values for $w_{sd}^{i}$ and $w_{td}^{i}$ are randomly assigned. Next, the assigned weights for both $T_{sd}$ and $T_{td}$ are divided by the summation of all weights and stored as $p_{t}$, as shown in Equation (3) [32]

(3) $$sum^{t}=\underset{w\in W^{t}}{\sum }w\;p^{t}=\left\{\frac{w}{sum^{t}}\;;w\in W^{t}\right\}$$

The training sample set $T=T_{sd}\cup T_{td}$ in ${Ω}_{s}^{'}$, $p_{t}$, and the test set S in ${Ω}_{t}^{'}$ are taken as input to the InstanceEasyTL algorithm (though note that S is not used for the updating of weights in each iteration of the algorithm, but rather just for determining the predicted class labels in the final iteration) and the expected class label of $h_{t}$ is calculated based on the intra-domain programming method (also called EasyTL) first detailed in [43].

Next, the error, $\epsilon_{t}$, between the predicted class labels, $h_{t}$, and the real class labels, y(x), is calculated via Equation (4) [32]

(4) $$\epsilon_{t}=\frac{1}{W_{t}}*\;\underset{\begin{array}{c} x\;\in T_{td}\\ w_{x}\in W_{td}^{t} \end{array}}{\sum }w_{x}\left|h_{t}\left(x\right)-y\left(x\right)\right|$$

The weights of $T_{sd}$ and $T_{td}$ are updated by the $\beta_{t}$-based function through Equation (5), detailed in [44]

(5) $$\beta_{t}=\epsilon_{t}/\left(1-\epsilon_{t}\right)\;\beta =1/\left(1+(2\ln n_{s}/N){\;}^{\frac{1}{2}}\right)$$

The weights are then updated via Equation (6) [32]

(6) $$W^{t+1}=\underset{\begin{array}{c} x\in T_{sd}\\ w_{x}\in W_{sd}^{t} \end{array}}{⋃}w_{x}\beta^{\left|h_{t}\left(x\right)-y\left(x\right)\right|}\;\cup \;\underset{\begin{array}{c} x\in T_{td}\\ w_{x}\in W_{td}^{t} \end{array}}{⋃}w_{x}\beta^{\left|h_{t}\left(x\right)-y\left(x\right)\right|}$$

These steps (Equation (3) through (6)) are repeated for N iterations. When t=N, the predicted class labels, $h_{f}\left(x\right)$, for the data in the test set, S, are calculated by Equation (7) [32]

(7) $$h_{f}\left(x\right)=\{\begin{array}{c} 1,\;\;\overset{N}{\underset{t=N/2}{\prod }}\beta_{t}^{-h_{t}\left[x\right]}\;\geq \;\overset{N}{\underset{t=N/2}{\prod }}\beta_{t}^{-1/2}\\ 0,\;otherwise\;\; \end{array}$$

## 4. Results

Table 1 shows the results of the mental workload level classification (Easy vs. Difficult) for all participants for the three classification paradigms. The cross-subject with transfer learning approach produced the best results with an average accuracy of 72.2% ± 5.3. This was 12.3% and 15.11% higher than the subject-specific and cross-subject without TL approach, respectively. A one-way ANOVA revealed a significant effect of the classification approach $\left(F_{2,34}=278.86;p<0.001\right)$, and post hoc Tukey–Kramer tests indicated these increases were both significant $\left(\left|t_{\left(68\right)}\right|>18.06;p<0.001\right)$. The result for the subject-specific approach was significantly higher than for the cross-subject without TL approach ($t_{\left(68\right)}=4.14;p<0.001$).

Table 2 shows the results of the affective state classification (Relaxed vs. Stressed) for all participants for the three classification paradigms. The best results again came from the cross-subject with transfer learning approach with an average accuracy of 74.2% ± 5.1, which exceeded the accuracy for the subject-specific approach by 9.78% and for the cross-subject without TL paradigm by 15.91%. A one-way ANOVA revealed a significant effect of the classification approach ($\left(F_{2,34}=154.95;p<0.001\right)$ and post hoc Tukey–Kramer tests revealed that these differences were both significant $\left(\left|t_{\left(68\right)}\right|>10.72;p<0.001\right)$. The result for subject-specific was significantly higher than cross-subject without TL approach $\left(t_{\left(68\right)}=6.73;p<0.001\right)$.

Since the classes are balanced for both the mental workload level classification and the affective state classification problems, the accuracy should be a valid measure of the classifier performance; however, the F1 score is given in Table 1 and Table 2 as well.

By the binomial test, the lower limit for statistical significance in a binary classification problem with *n* = 402, *p* = 0.5, and *α* = 0.05 is 54.2%. For the cross-subject with TL approach, accuracies for all subjects, for both classification problems, exceed this value by at least 9.2%.

**Figure 3:**
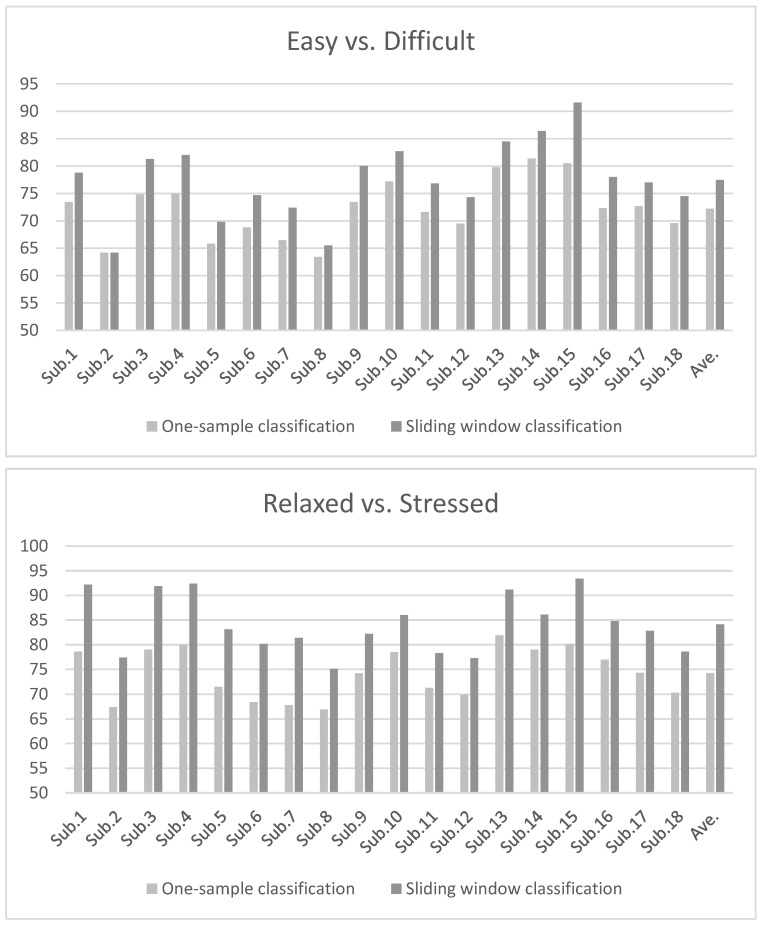
Classification accuracy of the cross-subject with TL approach with and without the application of sliding window classification.

To further improve online classification accuracy by reducing incorrect predictions resulting from sudden changes in the EEG signals, we applied a sliding window classification in which the final predicted class for each sample was determined by the majority vote of the output of the InstanceEasyTL classifier (i.e., the cross-subject with TL approach) for that sample and the two previous samples. This method results in a prediction every 2 s, the same as before. Using this sliding window classification method for the cross-subject with TL paradigm, the average accuracy for the Easy vs. Difficult classification significantly increased by 5.3% to 77.5% ± 6.9 $\left(t_{17}=-10.12;p<0.001\right)$ and the average accuracy for the Relaxed vs. Stressed classification significantly increased by 9.9% to 84.1% ± 5.9 $\left(t_{17}=-16.98;p<0.001\right)$ over all subjects. Figure 3 shows the classification accuracies using one-sample prediction as compared to the sliding window classification for all subjects.

**Figure 4:**
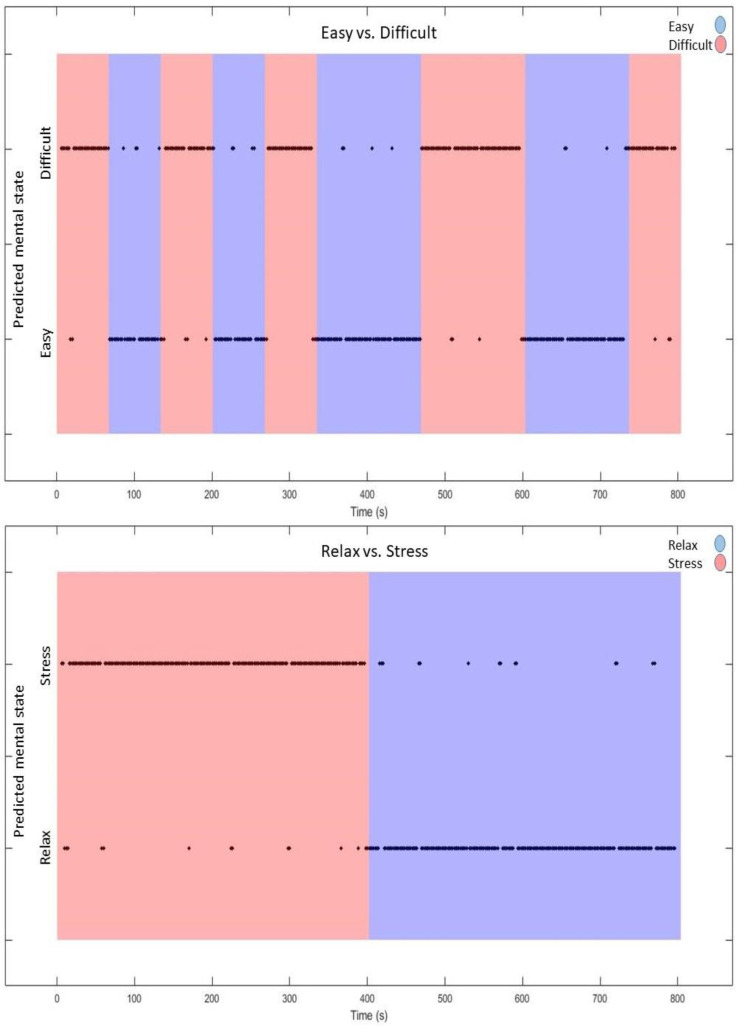
Simulated online output of the system for Subject 15 for both the Easy vs. Difficult and Relaxed vs. Stressed classification problems. After training the classifier on the first two blocks of data (combined with the data from all other subjects), consecutive samples from the final two blocks were classified (epochs were of length 4 s, with 2 s overlap). Classification was performed using the cross-subject with TL approach, with a sliding window classification over three samples. The shaded intervals indicate the actual mental state, while the black dots indicate the predicted state.

Figure 4 shows a simulation of online classification for one participant (Subject #15) for both the workload level and affective state classification problems. The figure shows the predicted class output using the sliding window classification every 2 s (recall classification was performed over 4 s epochs with a sliding window with 50% overlap) for the final two blocks of the session. The classifier for each individual sample was trained using the cross-subject with TL approach. As can be seen in the figure, the predicted classes follow the actual class labels with high accuracy—91.6% and 93.4% for workload level and affective state classification problems, respectively.

## 5. Discussion

The main objective of this study was to investigate the feasibility of performing simultaneous classification of mental workload and stress level in an online passive BCI. We investigated both subject-specific and cross-subject classification approaches, the latter with and without the application of a transfer learning technique called InstanceEasyTL [32] to align the distributions of data from the training and test subjects. In the subject-specific case, the first 50% of the individual’s data were used to train a regularized LDA classifier, and the final 50% of data were used as test data. For the cross-subject cases, the data from the 17 other participants were also added to the training set. Though performed offline, all steps of the analysis—including preprocessing, feature extraction, and classification—were conducted in a manner completely compatible with online implementation.

Our results showed that mental workload level (Easy vs. Difficult) and affective state (Relaxed vs. Stressed) could be classified in a manner suitable for online implementation with accuracies of 77.5% ± 6.9 and 84.1% ± 5.9, respectively, across 18 participants. Accuracies significantly exceeded chance (54.2% in this case) for all participants, for both classification problems. These results were achieved using cross-subject classification with transfer learning, which gave significantly better results than the other methods (i.e., entirely subject-specific or cross-subject without TL applied, with the same amount of training data taken from the test subject).

To the best of our knowledge at the time of writing, this study represents the first attempt to perform simultaneous classification of mental workload level and stress in an online passive BCI, though there are many studies that have looked at one or the other conditions individually through offline applications. Actually, online BCI studies are generally rather scarce. Recently, [48] presented an EEG-based classification of four mental states (fatigue, workload, distraction, and the normal state) for seven pilots in both offline and pseudo-online analyses. They proposed a multiple feature block-based convolutional neural network (MFB-CNN) with spatio-temporal EEG filters to recognize the pilot’s current mental states. In the pseudo-online analysis, they conducted an evaluation between one of the mental states (fatigue, workload, and distraction) and rest states and obtained the detection accuracy of 72%, 72%, and 61% for fatigue, workload, and distraction, respectively. In ref. [49], a novel strategy named adaptive subspace feature matching (ASFM) for cross-domain EEG-based emotion recognition was proposed. ASFM integrates both the marginal and conditional distributions. Both offline and online evaluations were performed, and the average classification accuracy of 75.1% ± 7.6 was achieved in the subject-to-subject evaluation for 15 subjects for the online analysis. It is worth mentioning that we employed the ASFM transfer learning algorithm to our data as well but did not achieve promising results. Even though it is difficult to directly compare our study to [48,49] due to differences in the experimental (e.g., type of task, number/length of trials) and analytical (e.g., preprocessing techniques, EEG features used, and classification algorithms) methods employed, the accuracies obtained are similar. The fact that our study involved a potentially much more complex scenario—that is, simultaneous classification of two states, where both states were confounding one another—makes our results even more encouraging.

Note that the InstanceEasyTL algorithm allowed us to achieve satisfactory classification accuracies despite a relatively small amount of training data taken from the test subject immediately prior to “online” classification. To simulate an online application of the system, the classifier was trained on the target subject’s first two blocks of data and tested on the subject’s final two blocks, to keep the data continuity in time. Therefore, about 13.5 min (12 trials × 67 s/trial) of training data were used from the test subject; that is a fairly reasonable length of time for system calibration prior to use. In the simulated online testing, about 402 epochs were tested one by one as time progressed. The time taken for data preprocessing, feature extraction, and classification of each 4-s epoch was 0.22, 0.59, and 0.03 s, respectively, for a total of less than 1 s (0.84 s) per epoch. All the algorithms were carried out using MATLAB R2019b with an Intel core i7-8th Gen processor.

Due to restrictions on research involving human participants arising due to the COVID-19 global pandemic, we were unable to conduct a separate experiment for online implementation and instead used the data previously collected and reported in [25,26] to simulate exactly an online classification scenario completely suitable for direct online implementation. Future work will involve realizing the cross-subject mental workload level and stress detection algorithms in an actual online application and evaluating them in more realistic, ecologically-valid task scenarios in which users experience different levels of workload and affective state.

## 6. Conclusions

In this study, we investigated the ability to classify both mental workload level and affective state simultaneously using methods appropriate for implementation in an online BCI. Using the InstanceEasyTL transfer learning algorithm proposed in [32], we achieved accuracies of 77.5% and 84.1% for mental workload level and affective state classification, respectively, using a database of “previous” subjects and just 13.5 min of training data from the test subject. Classification was performed every two seconds. These results are very promising and support the feasibility of developing a practical, online, passive BCI for use in realistic scenarios where both the cognitive and affective state of the user will be changing over time.

## Figures and Tables

**Table 1:** Mental workload level classification results.

	Mental Workload Level Classification Results (Easy vs. Difficult)					
	Subject-Specific		Cross-Subject without TL		Cross-Subject with TL	
Subjects	Accuracy	F1 Score	Accuracy	F1 Score	Accuracy	F1 Score
1	66.7	0.66	58.2	0.58	73.5	0.73
2	49.3	0.51	47.2	0.48	64.2	0.65
3	64	0.63	59.7	0.59	74.8	0.74
4	65.8	0.65	59	0.6	75.0	0.75
5	53.0	0.54	51.3	0.52	65.9	0.66
6	52.5	0.53	53.8	0.53	68.8	0.67
7	56.4	0.56	50.5	0.5	66.5	0.65
8	58.1	0.57	50.9	0.5	63.4	0.63
9	57.7	0.58	58.6	0.58	73.5	0.74
10	66.5	0.66	59.7	0.59	77.2	0.76
11	59.2	0.6	56.8	0.55	71.6	0.72
12	57.6	0.57	55.9	0.56	69.5	0.68
13	66.6	0.66	65.5	0.66	79.8	0.79
14	67.7	0.67	67.7	0.67	81.4	0.81
15	60.3	0.61	61.3	0.62	80.5	0.8
16	60.8	0.6	56.0	0.56	72.3	0.71
17	58.8	0.58	58.5	0.58	72.7	0.72
18	57.7	0.57	57.3	0.57	69.6	0.7
Mean:	59.9 ± 5.3	0.59 ± 0.04	57.1 ± 5.1	0.56 ± 0.05	72.2 ± 5.3	0.71 ± 0.05

**Table 2:** Affective state classification results.

	Affective State Classification Results (Relaxed vs. Stressed)					
	Subject-Specific		Cross-Subject without TL		Cross-Subject with TL	
Subjects	Accuracy	F1 Score	Accuracy	F1 Score	Accuracy	F1 Score
1	68.8	0.69	65.1	0.64	78.6	0.78
2	60.0	0.59	47.7	0.47	67.4	0.66
3	61.6	0.61	62.4	0.61	79.0	0.78
4	64.4	0.64	64.2	0.63	80.0	0.79
5	60.8	0.61	51.4	0.52	71.5	0.73
6	65.5	0.65	50.9	0.51	68.4	0.68
7	60.5	0.6	54.8	0.54	67.8	0.67
8	60.6	0.61	56.5	0.56	66.9	0.67
9	64.4	0.64	56.1	0.56	74.2	0.75
10	64.0	0.63	64.9	0.63	78.5	0.77
11	59.0	0.59	57.6	0.56	71.3	0.71
12	65.3	0.64	56.0	0.56	69.9	0.7
13	72.8	0.71	65.0	0.64	81.9	0.81
14	73.6	0.74	66.1	0.67	79.0	0.78
15	67.2	0.67	58.7	0.58	80.2	0.81
16	62.3	0.62	59.2	0.58	77.0	0.78
17	66.8	0.66	57.2	0.57	74.3	0.74
18	62.7	0.63	56.1	0.56	70.3	0.71
Mean:	64.5 ± 4.1	0.64 ± 0.04	58.3 ± 5.4	0.57 ± 0.05	74.2 ± 5.1	0.74 ± 0.05

## Data Availability

The data that support the findings of this study are available on request from the corresponding author.
